# Progress in Ophthalmology

**Published:** 1900-10-27

**Authors:** 


### Progress in Ophthalmology.

**The Operative Treatment of Strabismus.**[1]—Dr. A. E. Davis in a most exhaustive paper on the above subject comes to the following conclusions from the examination of a great number of cases:—

1. Hypermetropia and hypermetropic astigmatism are the causes of convergent strabismus in the great majority of cases.

2. As contributory causes may be mentioned (*a*) difference in acuteness of vision, either congenital or acquired, but usually acquired, and due to an unequal state of reproduction in the two eyes; (*b*) anything that interferes with the acuteness of vision, as opacities of the cornea, in the vitreous, or lens.

3. Faulty structure, insertion or innervation of the extrinsic muscles of the eye, may cause convergent strabismus; and paresis of the ciliary muscles has been given by some observers (Javal), also traumatism of the external rectus muscle at birth (Panas).

4. The amblyopia present in most cases of convergent strabismus is functional and acquired, and not congenital, except in rare cases.

The few examples of positive evidence on this point—that is, where cases have improved greatly in vision when the squinting eye was straightened, together with the cases where the amblyopia has been observed to develop after the strabismus begins—are of much more value than all the negative evidence in favour of the theory of congenital amblyopia.

5. The non-operative treatment of strabismus, atropin and the exclusion pad and (in patients old enough) glasses, the stereoscope, and bar reading, should be begun as soon as the squint is observed; for it is in the early cases that this form of treatment is capable of doing so much good. By means of it, if the case is taken in time, false fixation and the suppression of vision in the squinting eye is prevented, fusion of the images in the two eyes is encouraged, and form perception, that is, true binocular single vision, often maintained. Even where one or more of these functions have been lost, persistent effort in the non-operative methods of treatment frequently restores them.

6. That about 25 per cent. of cases of convergent strabismus may be cured by non-operative treatment.

7. Just as soon as the non-operative method of treatment ceases to improve the condition of the squint it is time to operate. Delay in operating after this is not only useless but harmful, because the habit of suppressing the image in the squinting eye becomes fixed and the amblyopia worse.

8. After the eyes have been operated on, the use of the stereoscope, bar reading, the pad, glasses and so forth, are of the utmost use in completing the cure, maintaining parallelism and establishing single binocular vision.

9. The "rational" treatment of strabismus is, as Priestly Smith says, "early treatment," and in every case the child should be thoroughly examined soon after the strabismus begins. This principle should be urged on the whole profession, in order that it may reach the public.

**The Differentiation of Ocular Affections commonly met with in Family Practice.**[2]—Dr. von Fleet, in a lecture on the above subject, says: We have, then, four conditions commonly met with in family practice, widely different as a rule both in symptoms and effects, and still so often simulating each other as to be difficult to properly separate, and he recapitualtes them:—

*Conjunctivitis.*—Burning, smarting and itching of eyelids; feeling of foreign bodies in the eye and discharge of pus; swelling and redness of the eyelids and conjunctiva; injection of the conjunctival blood-vessels; very little, if any, neuralgic pain, pupil of normal size, reacting to light; photophobia may or may not be present; tension normal.

*Keratitis.*—Increased flow of tears, injection of circumcorneal blood vessels; marked photophobia; more or less supra-orbital neuralgia; pupil contracted but dilates regularly under cocaine, which also relieves photophobia; tension normal or diminished; cornea may show an abrasion or an opacity.

*Iritis.*—Eyelids not affected except in intense cases, when they may be swollen; marked injection of circumcorneal blood vessels; no discharge as a rule, but when present is like the discharge of keratitis; pain marked and of the character of supra-orbital neuralgia; photophobia sometimes present, but not a marked symptom; cornea may appear hazy because of discoloration of iris; pupil contracted and dilates irregularly—if at all—under cocaine, tension slightly diminished.

*Glaucoma.*—Eyelids swollen in marked cases; no discharge as a rule, but watery when present; pain marked and of the same character as in iritis; pupil dilated; cornea may be hazy, but will be insensitive to touch, therein differing from keratitis and iritis, tension increased.

Having finished this recapitulation, he continues as follows: There are two forms of keratitis presenting symptoms which differ from those described as usually accompanying this affection; they are neuro-paralytic keratitis and keratitis due to malarial infection. Neuro-paralytic keratitis is ushered in with intense pain of a neuralgic character on the affected side, which on subsiding leaves diminished or entire loss of sensation of the parts supplied by the fifth nerve. You get in this stage an ulcerative keratitis, due to lowered vitality of the corneal tissue, which may end in total loss of the cornea. In this affection there is no photophobia, because the sensation of the cornea is lost. In malarial keratitis there is also a lack of sensitiveness in the cornea, without loss of sensation in the integument of the face, such as we find in neuro-paralytic keratitis. There is not the rapid destruction of corneal tissue in the malarial variety, although there is frequently an ulceration which resists the ordinary treatment, without producing permanent damage to the cornea.

Furthermore, in the diagnosis of iritis he [mentions a point which is usually not given in the books, but which he says is fairly constant in this affection. "By getting the patient to look in different directions and making gentle point pressure with a blunt instrument on the lids close to but outside of the limbus of the cornea, we detect somewhere an exquisitely tender point; this is found in nearly all cases of iritis, and is a valuable guide to diagnosis."

**Some Cases showing the use of Gas and Oxygen as an Anæsthetic in certain Ophthalmic Operations.** Dr. McCardie,[3] in a paper on this subject, quotes 11 cases where he used this anæsthetic, and says, as many of the operations are short ones, the use of gas and oxygen wherever possible will obviously have great advantages over the other two anæsthetics (chloroform and ether). In his remarks at the end he continues: "I have not yet had an opportunity of trying the mixture for iridectomy. Probably a deep enough anæsthesia for most eye operations is attainable in properly selected cases. Dexterous management is of course necessary in using the rather complex apparatus, and the more so as the face-piece and stopcock are so near the region of the operation. It will be observed that the age of the youngest patient was four years, and that of the oldest between sixty-five and seventy years."

The special advantages of using gas and oxygen in eye work are (1) the almost absolute safety of the mixture, which is the safest known; (2) the rapid induction of anæsthesia, the patient being under in less than two minutes; (3) the induction is without incident, and the maintenance in the best cases is quite sleep-like, after which full recovery takes place in less than two minutes; (4) the absence of after-effects, the patient being able to leave the room in a few minutes; (5) the eyeball refixed, the orbicularis relaxed, and congestion can be entirely avoided by the allowance of sufficient oxygen, while there is no retching, sickness, cough, straining, or unconscious after-movement causing increased intra-ocular vascular tension; (6) the upright chair or semi-recumbent position on a couch can be conveniently and safely assumed; (7) and this method of anæsthetisation is, for several of the above reasons, admirably adapted for consulting-room work.

With regard to limitations in usage, it is not judicious to give gas and oxygen to big, strong, or much-bearded men, to alcoholics of either sex, or to very young children for delicate operations. In adults especially it is important to keep the patient's head turned to one side to prevent obstruction to respiration, and in some cases the jaw must continuously be pushed forward to keep a free air-way. There is the disadvantage to the mixture that in operations immediately followed by pain the patient awakes at once to the sense of it, and it may be necessary to inject morphia. Most of these fourteen cases were tenotomies, the others being tumor of eyebrow, tumor of lid, scleral puncture for glaucoma, and one excision of eyeball.

**Pathological Relations of the Eyes and Teeth.**[4]—Dr. Lagleyze, of Buenos Ayres, has made an extended study of this subject, collating more than 100 separate papers bearing upon it, which have been written within the present century. He concludes that there are pathological relations between the teeth and the visual apparatus, but that some oculists have an exaggerated idea of the frequency of disturbances of vision due to dental disease. One should examine carefully the cavities immediately adjoining the orbit, and exclude diathetic and general conditions and disease of other distant organs, before advising radical dental operations. Even if a dental lesion exists it may be only coincident, or a trigeminal neuralgia may be felt in both the eye and the teeth.

Dental lesions may give rise to reflex disturbances of the visual apparatus, or inflammations may extend from the teeth to the orbit by continuity of tissue. All the teeth, although very rarely the lower, are capable of causing ocular disturbances. Periostitis and caries of the alveolus, and disease of the first molars and premolars of the upper jaw, are most likely to cause ocular lesions.

[1] Post-graduate, i. 1900. [2] Ditto. [3] Lancet, May 19, 1900. [4] Amer. Jour Med. Sci. March, 1900.

